# Improved adsorption reactions, kinetics and stability for model and therapeutic proteins immobilised on affinity resins

**DOI:** 10.1007/s10450-019-00106-5

**Published:** 2019-05-16

**Authors:** S. H. M. Hedberg, L. G. Brown, A. Meghdadi, D. R. Williams

**Affiliations:** 1 Surfaces and Particle Engineering Laboratory, Department of Chemical Engineering, Imperial College London, London, UK; 2 Present Address: Bioengineering Research Group, Department of Mechanical Engineering, University of Southampton, Southampton, England, UK

**Keywords:** Self-interaction chromatography, Affinity chromatography, Monoclonal antibodies, Polyclonal antibodies, Protein immobilisation, Coupling kinetics

## Abstract

Protein adsorption on solid state media is important for the industrial affinity chromatography of biotherapeutics and for preparing materials for self-interaction chromatography where fundamental protein solution thermodynamic properties are measured. The adsorption of three model proteins (lysozyme, catalase and BSA) and two antibodies (a monoclonal and a polyclonal antibody) have been investigated on commercial affinity chromatography media with different surface functionalities (Formyl, Tresyl and Amino). Both the extent of protein immobilised (mg protein/ml media) and the reaction kinetics are reported for a range of reaction conditions, including pH, differing buffers as well as the presence of secondary reactants (glutaraldehyde, sodium cyanoborohydride, EDC and NHS). Compared to the reaction conditions recommended by manufacturers as well as those reported in previous published work, significant increases in the extent of protein immobilisation and reaction kinetics are reported here. The addition of glutaraldehyde or sodium cyanoborohydride was found to be especially effective even when not directly needed for the adsorption to happen. For mAb and pIgG, immobilisation levels of 50 and 31 mg of protein/ml of resin respectively were achieved, which are 100% or more than previously reported. Enhanced levels were achieved for lysozyme of 120 mg/ml with very rapid reaction kinetics (< 1 h) with sodium cyanoborohydride. It can be concluded that specific chromatography resins with Tresyl activated support offered enhanced levels of protein immobilisation due to their ability to react to form amine or thio-ether linkages with proteins. Additionally, glutaraldehyde can result in higher immobilisation levels whilst it can also accelerate immobilisation reaction kinetics.

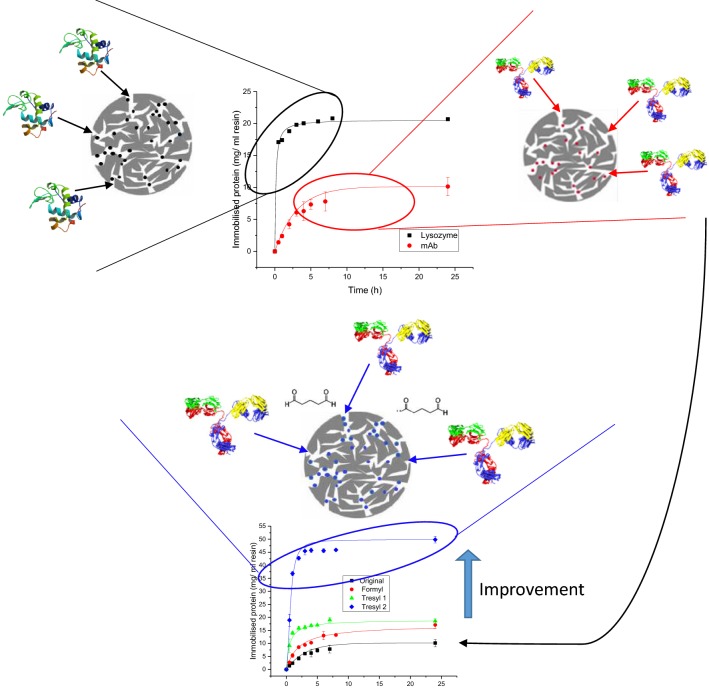

## Introduction

One of the fastest growing sectors in the pharmaceutical industry is the production of protein based therapeutics that have been shown to be successful in treatments of cancer, heart disease, diabetes, autoimmune diseases and infectious diseases (Pavlou and Reichert 2004). Antibody therapeutics are part of an important class within the biopharmaceutical industry, especially, since monoclonal antibodies have been used for many therapeutic conditions (Shukla and Kandula 2009). Despite their success in treatments and the growing numbers of protein based pharmaceuticals, the manufacture of these proteins in a cost-effective and reliable way remains a challenge (Hernandez 2015; Shukla and Gottschalk 2013).

A major issue in the manufacture of protein therapeutics is the formation of protein aggregates, potentially in all stages of the process (Chi et al. 2003). The formation of aggregates can lead to a loss of bioactivity and function of the products, which could lead to an immunogenic reaction or adverse effects (Rosenberg 2006). Therefore, the ability to predict, restrict or minimise aggregation would still be crucial for a successful manufacturing or formulation process (Vázquez-Rey and Lang 2011).

Protein–protein interactions are known to be involved in the aggregation behaviour of proteins in solutions (Wang 2005; Durbin and Feher 1996). A widely used predictive method to study protein–protein interactions, protein phase behaviour and colloidal stability is the use of the osmotic second virial coefficient (B_22_), a physicochemical property that can also be applied to protein behaviour in solution (George and Wilson 1994; Coen et al. 1995; Curtis et al. 2001; Lewus et al. 2011; Rakel et al. 2013; Hedberg et al. 2018a). Self-Interaction chromatography (SIC) has lately become an attractive technique to determine B_22_ values due to the automation and the speed of the measurements (Wilson and DeLucas 2014).

### Self-interaction chromatography

SIC is based on the retention interactions between protein molecules in solution and the same protein which is adsorbed to solid state resin particles and forms the stationary phase in this chromatography method. The protein immobilisation procedure has long been considered a bottleneck of SIC (Bajaj et al. 2007), due to the experiments being non-automated as well as requiring relatively substantial amounts of proteins. However, studies has shown that the immobilisation requirements can be reduced with cross-interaction chromatography (Jacobs et al. 2010; Hedberg et al. 2018b) or can be done with minimal amounts of protein using both microfluidic chips and micro columns (García et al. 2003; Deshpande et al. 2009; Martin and Lenhoff 2011; Hedberg et al. 2016).

Therefore, a critical aspect in SIC revolves around the choice and use of the stationary phase resin as well as the solution conditions in terms of adsorption chemistry (Rakel et al. 2013; Hedberg et al. 2015). This is particularly important in the immobilisation of therapeutic proteins such as mAbs that may have high costs. An improved understanding of the immobilisation process will allow a specific amount of protein to be employed to achieve a specific required surface coverage, with minimal amounts of the original protein being lost during washes and experimental runs. Furthermore, knowledge of the kinetics of the adsorption of the proteins to specific chromatography resins will allow the time used for the immobilisation to be optimised. This optimisation could in turn make the immobilisation process faster and decrease the time needed for the first B_22_ values to be measured.

Many of these factors are drivers for a better controlled immobilisation process, which is currently the one most challenging part in SIC. Surprisingly few papers quantify the precise amount of protein immobilised, including many pioneering papers with one notable exception (Tessier et al. 2002a). This deficiency is an important limitation for researchers who need to know how well an immobilisation procedure works and if it is suitable for SIC. Furthermore, no researchers have reported on the kinetics of the immobilisation reaction, so the time needed to complete the immobilisation is often unknown.

### Other use of immobilisation

In fields other than SIC, there have been many studies on how to optimise functional activities of proteins and strategies chosen for immobilisation (Steen Redeker et al. 2013). Similarly, kinetics of protein coupling to different types of particles or resins have been reported (Blanchette et al. 2010; Zhu and Carta 2016; Heck et al. 2014).

Apart from SIC, the immobilisation of functional proteins and enzymes onto solid supports has also shown to be useful in other areas including clinical diagnostics (Ispas et al. 2012), industrial bio-catalysis for green chemicals manufacture (Sheldon and van Pelt 2013), food safety and environmental monitoring (Heck et al. 2014; Amine et al. 2006).

Additionally, efficient capture of therapeutic proteins is also of high importance in downstream purification and there have been efforts into optimising multimodal resins reaction conditions to capture mAbs (Pinto et al. 2016) or to separate proteins using ion affinity chromatic processes (Sharma and Agarwal 2002).

In this paper the kinetics of the protein immobilisation process are studied across a range of standard and optimised solution conditions using a series of commercial affinity resins. The reaction conditions studied were chosen carefully based on the manufacturer’s recommendations for the chromatographic resins as well as the solution conditions previously reported to be successful for immobilisation of other proteins. Due to the vast number of conditions and immobilisation procedures possible, this paper focuses on the three commonly used affinity resins, with commonly used buffer solutions and reagents.

### SIC immobilisation procedures

SIC has been reported by many researchers who have employed the Toyopearl AF-650M resins in the Formyl, Amino and Tresyl forms. There are also a small number of papers that have reported pre-packed Hi-Trap NHS-activated columns (Rakel et al. 2013; Jacobs et al. 2010) or periodate-activated agarose (Teske et al. 2004). The Toyopearl 650 M resins have been studied in detail by DePhillips and Lenhoff (2000), reporting useful parameters particularly applicable in determining the B_22_ in SIC, making these resins especially favoured by researchers.

The Toyopearl AF-650M resins are porous affinity resins with an average particle size of 65 µm with 1000 Å pore diameters. All of these resins consist of a hydrophilic polymer matrix, cross-linked polymethacrylate, with different surface groups depending on the type of resin. Toyopearl AF-Formyl is a reactive resin that has aldehyde surface groups and can bind to amine groups on the protein. Toyopearl AF-Amino has amine groups on the surface instead and can bind to carboxyl groups on the proteins via peptide bond formation or aldehyde groups through reductive amination. Toyopearl AF-Tresyl is an activated resin and is highly reactive towards amine and thiol groups (Nakamura et al. 1989). These resins will be referred to in a short form as Formyl, Amino or Tresyl in this work.

The second most important factor is the selection of reaction solution conditions that are suitable for both the resins themselves and the proteins in the immobilisation procedure. In addition, some of these immobilisation reactions require the use of coupling agents.

It has been generally seen in the literature that relatively little variation to the immobilisation procedure has been reported for model proteins such as lysozyme, catalase and BSA. Many of the papers listed in Table 1, which report new SIC experiments, employ the same immobilisation techniques. This paper considers two model proteins as well as a therapeutic monoclonal antibody (mAb) and a polyclonal antibody (pIgG).

**Table 1:** List of papers immobilising lysozyme, catalase, BSA and mAbs on Toyopearl AF-resins

Protein	Resin used	Condition	Coupling agent	After immobilisation 1. Washing step 2. Capping reaction step	References
Lysozyme	Formyl	0.1 M potassium phosphate pH 7.5	NaBH_3_CN	1. Same buffer wash 2. 1 M MEA pH 8 + NaBH_3_CN	Tessier et al. (2002a)
Lysozyme	Formyl	0.1 M potassium phosphate pH 7.0	NaBH_3_CN	1. 0.1 M acetate pH 4.5 wash 2. 1 M MEA pH 8 + NaBH_3_CN	Valente et al. (2005)
Lysozyme	Formyl	0.1 M potassium phosphate pH 7.0	NaBH_3_CN	1. Phosphate buffer wash 2. 1 M MEA pH 8 + NaBH_3_CN	Johnson et al. (2009)
Lysozyme	Formyl	0.1 M potassium phosphate pH 7.5	NaBH_3_CN	1. Same buffer wash 2. 1 M MEA pH 8 + NaBH_3_CN	Le Brun et al. (2010a)
Lysozyme	Formyl	0.1 M potassium phosphate pH 7.5	NaBH_3_CN	1. Same buffer wash 2. 1 M MEA pH 8 + NaBH_3_CN	Quigley et al. (2013)
BSA	Amino	1 M potassium phosphate pH 8.5	Glutaraldehyde	1. Same buffer wash 2. 1 M MEA pH 8	Tessier et al. (2002b)
BSA	Amino	1 M potassium phosphate pH 8.5	Glutaraldehyde	1. Same buffer wash 2. 1 M MEA pH 8	Dumetz et al. (2007)
Catalase	Amino	5 mM MES, 0.1 M NaCl pH 6.5	EDC & NHS	DI Water wash	Dumetz et al. (2007)
Catalase	Amino	5 mM MES, 0.1 M NaCl pH 6.5	EDC & NHS	Not specified	Quigley and Williams (2015)
mAb	Tresyl	0.1 M NaHCO_3_, 0.5 M NaCl pH 8.1	None	0.1 M Tris/HCl, 0.5 M NaCl pH 8.1	Jacobs et al. (2010)
mAb (IgG1)	Formyl	0.1 M potassium phosphate pH 7.5	NaBH_3_CN	1. Same buffer wash 2. 1 M MEA pH 8 + NaBH_3_CN	Le Brun et al. (2010b)
mAb (IgG1)	Amino	5 mM K_2_HPO_4_, 0.25 M NaCl pH 8.0	Glutaraldehyde	–	Lewus et al. (2011)
mAb	Formyl	0.1 M potassium phosphate pH 7.5	NaBH_3_CN	1. Same buffer wash 2. 1 M MEA pH 8 + NaBH_3_CN	Binabaji et al. (2013)

*NaBH*_*3*_*CN* sodium cyanoborohydride, *MES* 2-(N-morpholino)ethanesulfonic acid, *EDC N*-(3-dimethylaminopropyl)-*N*′-ethylcarbodiimide hydrochloride, *NHS* *N*-hydroxysuccinimide, *TRIS* tris(hydroxymethyl)aminomethane, *MEA* monoethanol amine

Many of these buffer solutions and coupling agents have been carefully selected in accordance with manufacturer’s guidelines, which have been specified in Table 2.

**Table 2:** Protein coupling densities for Toyopearl media (Chromatographic Process Media Catalogue, Tosoh Bioscience)

Resin media	Formyl (mg/ml resin)	Amino (mg/ml resin)	Tresyl (mg/ml resin)
Protein coupled			
BSA	14	19.2	12.4
Catalase	–	–	–
Lysozyme	20	5.8	60
mAb (IgG1)	–	–	–
Human IgG	15	6.7	10
Coupling agent	NaBH_3_CN	NaBH_3_CN/Carbodiimide	Not required
Optimal pH	6.9–9.0	4.5–6.0	7.0–9.0

Table 2 summarises the resin manufacturer’s recommendations of suitable buffer conditions and coupling agents that can be applied to the Toyopearl resins discussed here. The table estimates the amounts of protein which can be immobilised on each resin in mg of protein per ml of resin.

## Materials and methods

### Materials

These experiments used hen egg white lysozyme (crystalline white powder, EC 3.2.1.17), bovine liver catalase (lyophilised powder, EC 1.11.1.6), Bovine Serum Albumin (BSA), (lyophilised powder, A-7638) and IgG from human serum (lyophilised powder, A-4506), all obtained from Sigma-Aldrich (Dorset, UK). Additionally, an IgG1 type monoclonal antibody was supplied by Fujifilm Diosynth Biotechnologies which was highly purified (pI of 8.6 and molecular weight 144.5 kDa). Potassium phosphate, dibasic and monobasic sodium phosphate, sodium chloride, sodium bicarbonate, MES trihydrate, hydrochloric acid (HCl), sodium cyanoborohydride (NaBH_3_CN), ethanolamine (MEA), *N*-(3-Dimethylaminopropyl)-*N*′-ethylcarbodiimide hydrochloride (EDC), *N*-Hydroxysuccinimide (NHS) and glutaraldehyde were all purchased from Sigma-Aldrich (ACS grade or BioXtra grade). Sodium hydroxide and acetone were obtained from VWR (Leicestershire, UK). Toyopearl AF-Formyl-650 M (808004), Toyopearl AF-Amino-650 M (808002) and Toyopearl AF-Tresyl-650 M (814471) media were purchased from Sigma-Aldrich. For buffer preparation ultrapure deionised water (resistivity 18.2 MΩ∙cm) was used. The pH of the buffers were adjusted with HCl or NaOH and monitored using a Mettler Toledo FiveEasy pH meter. All solutions were filtered using 0.22 µm bottle-top filters from Millipore in order to remove particulates.

### Immobilisation

The immobilisation of the proteins were all performed in a similar fashion on Toyopearl AF particles together with recommended buffer solutions and appropriate other reagents. 0.3 to 1 ml of these particles were first washed with ultrapure deionised water (Elga Centra) in at least 30–50 times the particle volume, then centrifuged and re-suspended in water three times. The particles were then mixed with protein in the selected buffer at a suitable concentration so the total solution volume is three to four times the particle volume. Afterwards, the requisite coupling agent was added to the particle and protein suspension, at the following concentrations per ml of resins; NaBH_3_CN 17 mg, EDC 75 mg and NHS 5 mg. In the case of glutaraldehyde, the resin was activated firstly with 1 ml glutaraldehyde then washed extensively (at least 150 times the resin volume recommended) and, finally, the protein solution was added. The reaction was left to proceed on a rotary mixer at room temperature; 21 °C ± 1 °C. Samples were taken before reaction started (0 h) and approximately every hour (until 8 h) and after overnight reaction (24 h). The next day the particles were washed with the previously used buffer and the remaining active sites were capped in a number of different ways depending on the immobilisation method used. AF-Formyl with NaBH_3_CN was capped using 17 mg of NaBH_3_CN and 5 ml of 1 M MEA pH 8 per ml of resin and added to the rotary mixer for 4 h. AF-Tresyl particles were mixed with 0.1 M Tris–HCL solution at pH 8 and placed on the rotary mixer overnight. Samples were taken after the wash and capping reaction in order to determine protein loss. The protein concentration of the samples was measured using UV/Vis spectroscopy using a NanoDrop 2000 (Thermo Scientific) at 280 nm and selected samples were verified using a BCA Assay from Thermo Scientific Pierce.

## Results and discussion

The selection of optimal immobilisation conditions and resins is not trivial and is based on careful design of previously successful combinations. The manufacturer suggests pH conditions where the specific resins would perform the best, however, this condition would also need to match the conditions where the specific protein of interest would be the most stable with the salt types and concentrations selected.

When investigating the protein purity before and after the immobilisation, it was concluded that there is a higher possibility that the protein primarily binds to the resin whilst in its monomeric form. This was found both due to higher immobilisation efficiency for more purified samples (in the case of mAbs) as well as due to the aggregation measurements taken before and after immobilisation. The protein remaining after immobilisation has a higher concentration of aggregates, which approximates in total to the amount of aggregated protein left in the original solution after the amount bound on the resin is considered. So, unless the immobilisation process induced the formation of aggregates, this indicates that the protein is most likely to immobilise only when stable and in its monomeric form. As indicated by Tessier et al. (2002a) when calculating B_22_ it is assumed that all proteins immobilise in random orientations on the resin.

To normalise the data reported here all the results are expressed in terms of mg of protein/ml of resin. The results are based on n ≥ 3 samples with standard deviation (s.d.) reported. Tresyl is in solid form instead of slurry or gel form so there is a need to use a conversion for the expression mg/ml of resin. The conversion used here is 1 g of Tresyl yields 3.5 ml of resin slurry as suggested by the manufacturer.

### Original immobilisation conditions

The original conditions reported here are those that have been the most commonly published based on the papers listed in Table 1. Table 1 shows that catalase and BSA have been immobilised on the Amino resin whilst lysozyme and monoclonal antibodies have been immobilised on the Formyl resin. Several conditions used in publications detailed in Table 1 follow the recommendations by the manufacturer presented in Table 2.

**Fig. 1:**
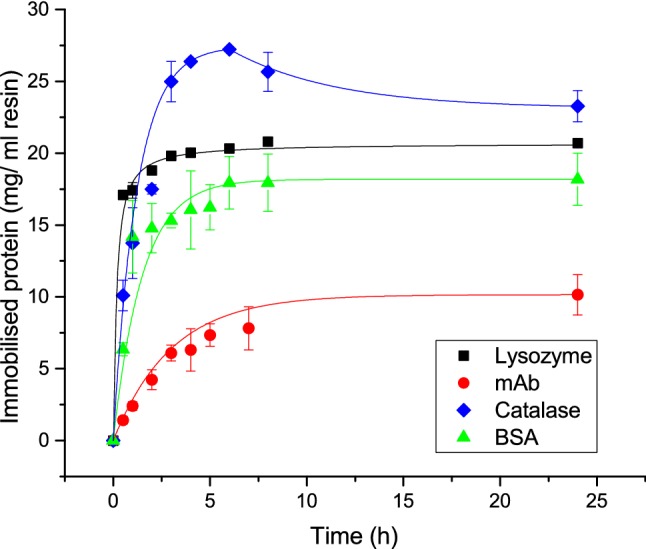
Immobilisation kinetics for BSA and catalase on Toyopearl AF-Amino particles and lysozyme and mAb on Toyopearl AF-Formyl particles; filled square: Immobilisation of lysozyme using 0.1 M potassium phosphate pH 7.5 with NaBH_3_CN; red filled circle: Immobilisation of mAb using 0.1 M potassium phosphate pH 7.5 with NaBH_3_CN; green filled triangle: Immobilisation of BSA using 1 M potassium phosphate pH 8.5 with glutaraldehyde; blue diamond: Immobilisation of catalase using 5 mM MES, 0.1 M NaCl pH 6.5 with EDC and NHS

Figure 1 shows the results obtained when immobilising BSA and catalase on the amino resin and lysozyme and mAb on the Formyl resin. Catalase was the most successful immobilisation of approximately 23–27 ± 1 mg followed by lysozyme with 20 mg/ml and BSA with 18 ± 2 mg immobilised. Least successful is the immobilisation of mAb that only manages to adsorb 9 ± 2 mg.

Catalase reaches its maximum immobilisation density at 6 h and after a slight decrease is observed. The changes in catalase adsorption may be due to fluctuations in protein concentration from 4 to 8 h and 24 h, where the 24-h samples would be more homogeneously mixed after being left overnight on a rotary mixer and arguably the most reliable immobilisation result. The amount of lysozyme immobilised corresponds very well with the amount reported by the manufacturer for lysozyme using Formyl particles. The results seem to be robust and produced minimum variation between the replicate samples and the repeats of the experiment, resulting in minimum variability (s.d.). Using identical reaction conditions for the mAb as for lysozyme, only 9 ± 2 mg was immobilised. Therefore, less than half the amount is immobilised for mAb compared to lysozyme and is more than a third lower than the amount reported by the manufacturer, albeit at slightly differing reaction conditions.

Most of these immobilisation reactions reach the maximum coverage after approximately 6 h, except for mAb, which takes at least 24 h.

### New optimised immobilisation conditions for BSA, catalase and lysozyme

Based on the results obtained from the initial experiments presented in Sect. 3.1, a select number of new reaction conditions were tested to improve the immobilisation efficiency of the proteins. These variables included varying the resins, buffer conditions and coupling agents according to the manufacturer’s specifications (Table 2) and as recommended in the literature (Table 1).

Immobilisation of BSA can be performed in principle according to the conditions outlined in Table 2 using all 3 resins, with Amino as the preferred media. The first optimisation experiment tested all 3 resins using reaction conditions previously successful for other proteins such as catalase and lysozyme in Table 1. This involved also testing BSA using the Amino resin with EDC and NHS, previously used for catalase and with Tresyl resin that according to Table 2 has a high immobilisation efficiency for many proteins.

**Fig. 2:**
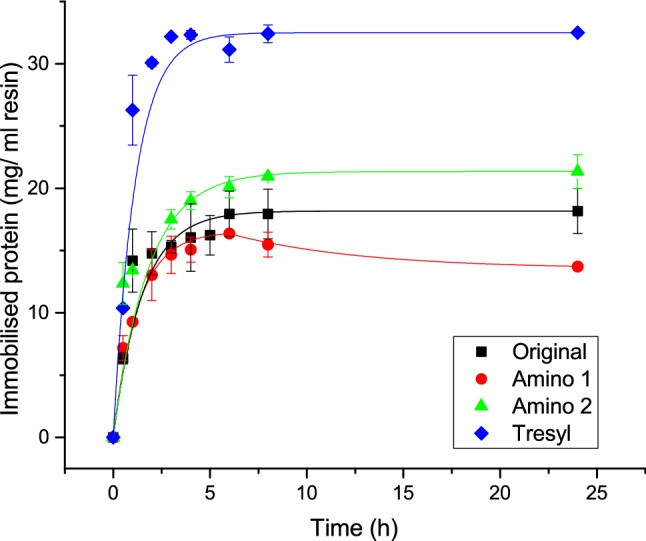
Immobilisation kinetics for BSA; filled square: Original immobilisation condition on Amino particles using 1 M potassium phosphate pH 8.5 with glutaraldehyde; ref filled circle: Immobilisation on Amino particles using 5 mM MES pH 5.5 with EDC and NHS; green triangle: Immobilisation on Amino particles using 5 mM MES, 0.1 M NaCl pH 6.5 with EDC and NHS; blue diamond: Immobilisation on Toyopearl AF-Tresyl particles using 1 M potassium phosphate pH 8.5 with glutaraldehyde

Figure 2 displays the original BSA immobilisation from Fig. 1 (filled square) and three new, optimised conditions, not previously used for BSA immobilisation. Another immobilisation condition for BSA, using Formyl resins with NaCNBH_3_ was not successful as only 5 mg was adsorbed at most, and before decreasing again with time; it is not shown in Fig. 2. Figure 2 shows that the results for BSA could be successfully improved using the Tresyl resin instead of Amino with added glutaraldehyde (blue diamond), which were the most efficient conditions with 32 ± 1 mg immobilised. It could also be improved slightly relative to the original conditions with MES pH 6.5 combined with EDC & NHS where 21 ± 1 mg was immobilised however MES pH 5.5 with EDC and NHS resulted in significantly lower amount of BSA adsorbed. In the case of Amino only one immobilisation reaction can occur with the carboxyl functional groups on the protein surface, whilst for Tresyl both thiol and amine groups on the protein surface can react. The kinetics of using Tresyl resins instead of Amino have shown to not only increase the immobilisation density significantly but also speed it up. Within an hour the immobilisation with Tresyl has improved significantly in comparison to the other adsorption reactions, enabling critical time to be saved.

Immobilisation of catalase was optimised in a comparable way using similar conditions as the ones used for BSA, with a number of alternative buffers for Amino and one for Formyl.

**Fig. 3:**
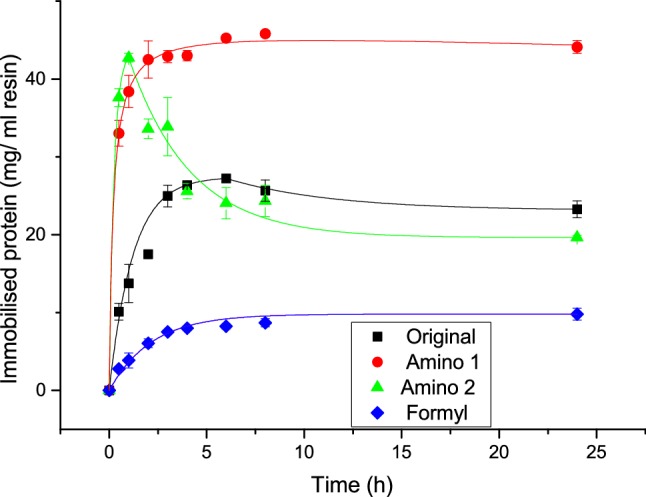
Immobilisation kinetics for catalase; filled square: Original immobilisation condition on Amino particles using 5 mM MES, 0.1 M NaCl pH 6.5 with EDC and NHS; red filled circle: Immobilisation on Amino particles using 5 mM MES pH 5.5 with EDC and NHS; green triangle: Immobilisation on Amino particles using 1 M potassium phosphate pH 8.5 with glutaraldehyde; blue diamond: Immobilisation on Formyl particles using 0.1 M potassium phosphate pH 7.5 with NaBH_3_CN

Figure 3 shows the improved reaction conditions for catalase. The original condition (filled square) is shown to be quite efficient but can be further optimised by lowering the pH to 5.5 and reducing the salt concentration using the same coupling agents. This condition turns out to be very close to the isoelectric point (pI) of pH 5.4. Unlike BSA, catalase immobilisation does not stabilise when adding glutaraldehyde (green triangle) as the amount of protein immobilised starts decaying after an hour quite dramatically. This effect is most likely due to the instability of catalase in the buffer condition combined with glutaraldehyde and not due to concentration fluctuations previously seen for catalase in the original condition and BSA in MES pH 5.5. This observation led to the assumption that the same reaction condition for Tresyl was also unlikely to be successful. The formyl resins can be used for catalase but with a minimal efficiency, which is most probably directly limited by the number of available amine group binding sites on the protein surface. In this case, the immobilisation reaction is not only allowing the amount immobilised to be increased but also dramatically shortening the reaction time as only half an hour was needed to improve the full immobilisation efficiency.

In the case of the immobilisation of lysozyme, Tresyl was the obvious choice with different buffer combinations based on the manufacturer’s recommendations (Table 2).

**Fig. 4:**
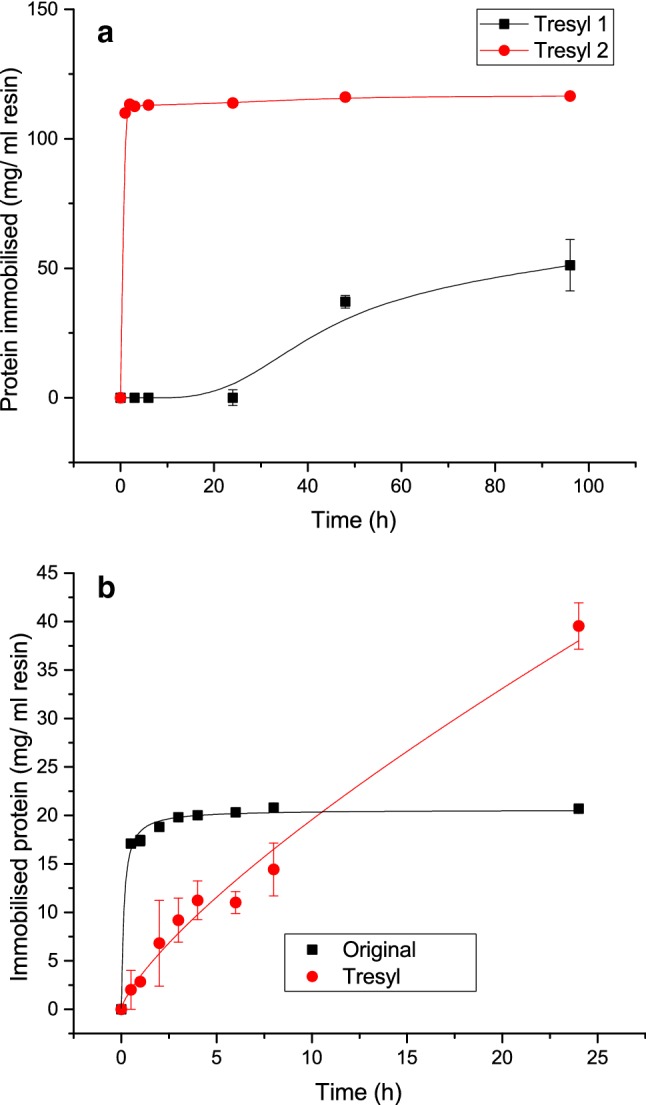
**a** Immobilisation kinetics for lysozyme; filled square: Original immobilisation condition on Formyl particles using 0.1 M potassium phosphate pH 7.5 with NaBH_3_CN; red filled circle Immobilisation on Tresyl particles using 0.1 M potassium phosphate pH 7.5 with NaBH_3_CN. **b** Immobilisation of lysozyme over a longer time span; filled square: Immobilisation on Tresyl particles using 0.1 M potassium phosphate pH 7.5 (without NaBH_3_CN); red filled circle: Immobilisation on Tresyl particles using 0.1 M potassium phosphate pH 7.5 with twice the original amount of NaBH_3_CN

Figure 4a displays one improved condition for lysozyme using Tresyl with the same buffer condition and coupling agent as the original condition with Formyl. Here, lysozyme is seen to immobilise very slowly onto Tresyl but this dramatically increases between 8 and 24 h. Tresyl immobilisation was thus investigated over a longer time period up to 96 h, as shown in Fig. 4b. This figure shows that the immobilisation of lysozyme on Tresyl particles over 4 days where significant changes in the kinetics are observed. Lysozyme immobilised on Tresyl in phosphate buffer with no coupling agent will immobilise slowly and will not be fully immobilised even in 4 days. If the amount of coupling agent is approximately doubled instead it looks very similar to the other kinetic profiles seen with the sharpest increase in the beginning and fully immobilised after a few hours. Comparing (red filled circle) in Figs. 4a with (red filled circle) and (filled square) in 4b it can directly be seen what effect NaBH_3_CN has on lysozyme immobilisation on Tresyl particles. This enhanced level of immobilisation and faster kinetics clearly show a significant catalytic effect with increased levels of NaBH_3_CN, as the concentration directly increases the reaction rate as well as the amount of protein adsorbed. Without NaBH_3_CN, the full immobilisation procedure would take several days, which is not ideal. The addition of NaBH_3_CN speeds up the reaction ≥ 6 times, with the reaction rate dependent on the concentration of NaBH_3_CN. Similar immobilisation efficiency can be achieved without the addition of any NaBH_3_CN, though the reaction is longer.

Immobilisation of lysozyme has even been performed using other buffer conditions and a useful condition that does not require any addition of NaBH_3_CN is Tresyl with 0.1 M sodium hydrogen carbonate and 0.5 M NaCl pH 8.1 similar to what has been used for mAb immobilisation. This condition has yielded around 110-120 mg of lysozyme/ml tresyl immobilised in 48 h, similar to (red filled circle), but with a slightly slower reaction rate.

### Optimised immobilisation of a monoclonal antibody and a polyclonal antibody

The same experimental approach as used for the model proteins was also applied to the monoclonal and polyclonal antibodies. The monoclonal antibody (mAb) was primarily immobilised using Formyl, Tresyl and Amino using similar conditions as tested for the model proteins.

**Fig. 5:**
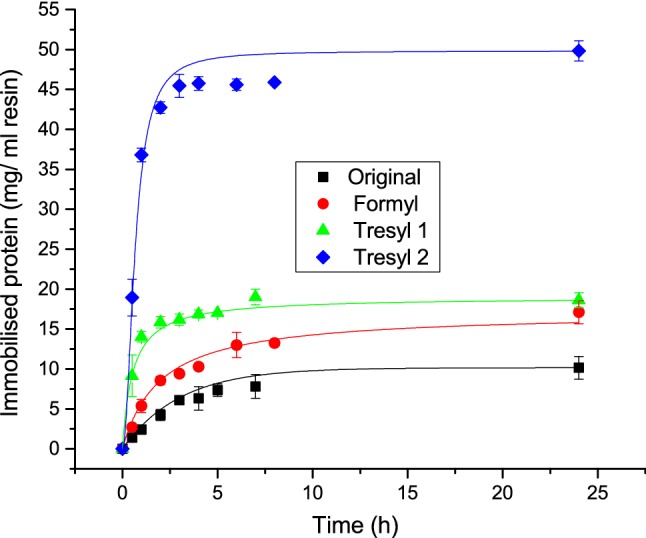
Immobilisation kinetics for mAb; filled square: Original immobilisation condition on Formyl particles using 0.1 M potassium phosphate pH 7.5 with NaBH_3_CN; red filled circle: Immobilisation on Formyl particles using 0.1 M sodium phosphate pH 6.9 with NaBH_3_CN; green triangle: Immobilisation on Tresyl particles using 0.1 M NaHCO_3_, 0.5 M NaCl pH 8.1; blue diamond: Immobilisation on Tresyl particles using 0.1 M NaHCO_3_, 0.5 M NaCl pH 7.5 with glutaraldehyde

In Fig. 5 it can be seen that the original (filled square) immobilisation condition is the least efficient condition reported here. When changing the buffer conditions closer to the formulation condition of pH 6.9, allowing a higher net positive charge on the mAb, it is seen that the amount immobilised is increased by 100% (red filled circle). A similar slightly improved level of immobilisation is seen if the resin is changed to Tresyl 1 (green triangle) and the immobilisation procedure follows (Jacobs et al. 2010) in Table 1. Additionally, this Tresyl 2 condition (blue diamond) was substantially improved by adding glutaraldehyde that had shown successes for BSA in Fig. 3 and as previously reported (Lewus et al. 2011). This condition was shown to be the best with an increase of 500% from the original experimental conditions when a maximum of 48 ± 1 mg of protein was immobilised. This immobilisation method did not work for the mAb with Amino resins. Using citrate buffer pH 5 with EDC and NHS yielded < 1 mg/ml immobilised and is therefore not reported.

As a polyclonal antibody (pIgG) is similar to a mAb apart from particular regions where the structure is changed, the conditions applied in this case were the best Formyl and the best Tresyl conditions for the mAb.

**Fig. 6:**
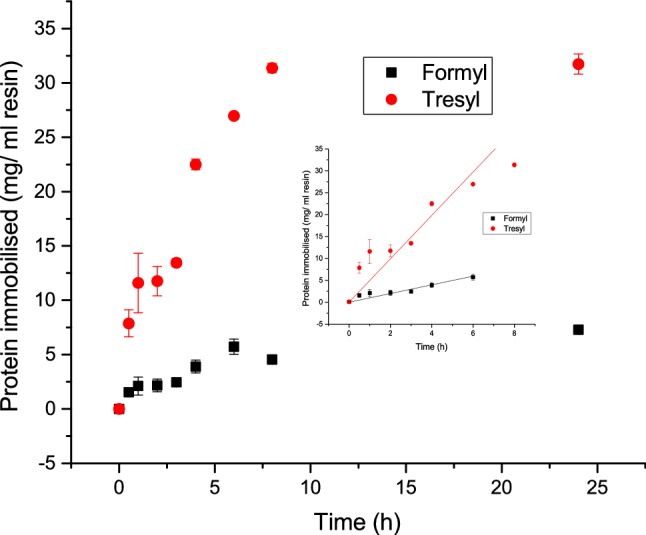
Immobilisation kinetics for pIgG; filled square: Immobilisation on Formyl particles using 0.1 M sodium phosphate pH 6.9 with NaBH_3_CN; red filled circle: Immobilisation on Tresyl particles using 0.1 M NaHCO_3_, 0.5 M NaCl pH 7.5 with glutaraldehyde

Figure 6 shows two of the most promising conditions tested for mAb, applied for the immobilisation of the pIgG. The pIgG was shown to be more difficult to immobilise, however, the same condition Tresyl with glutaraldehyde was the most efficient one to use with 31.5 ± 1 mg protein immobilised/ml resin. Again the Tresyl showed vastly superior amounts adsorbed.

### Dependence on pH and wash solutions

pH is a potentially important factor for optimising immobilisation conditions as indicated in Table 2. The impact of the change of pH for immobilisation conditions was investigated here and has been reported by other researchers (Rakel et al. 2013). The pH range showed to have minimal effect on lysozyme and mAb using Formyl particles between pH 6.5 and 8.0, with pH 7.5 the most preferable condition for lysozyme and pH 8.0 the most preferable condition for the mAb. The maximum amount immobilised was 20.7 mg protein/ml resin for lysozyme and 13.9 mg protein/ml resin for mAb with the variability between the highest and lowest 1.7 mg for lysozyme and 3.8 mg for mAb.

As referenced in Table 1, many researchers exposed their resin-protein beads to various washing solutions and MEA capping conditions after the immobilisations. Some of these conditions were shown not to be recommended for certain types of immobilisation conditions as they de-coupled a significant amount of protein. Capping with MEA has been widely used in terms of the immobilisation of lysozyme and BSA on Formyl or Amino resins respectively. However, the washing and MEA capping have shown less desirable for mAb immobilisation on Formyl as it can lead to a loss of ≥ 5% of the final amount immobilised and for pIgG almost 25% of the amount of protein coupled was lost. The combination of MEA capping using Tresyl resins was shown not to be a good combination, worst being BSA with approximately a 35% (± 6%) loss of protein after immobilisation. The recommended capping procedure for Tresyl resin is instead Tris/HCl which causes minimal losses of protein.

### Immobilisation kinetics

**Fig. 7:**
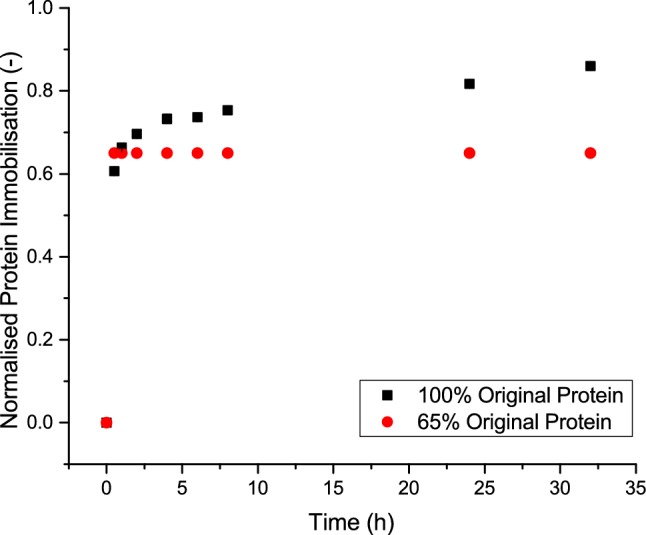
Immobilisation of lysozyme over time for different concentrations of proteins using formyl with 0.1 M potassium phosphate buffer pH 7.5 with full amount of protein (> 20 mg/ml of resin) and a reduced amount (65% of full)

Apart from that the immobilisation efficiency that is improved, the speed at which the reaction is occurring has been dramatically increased. The importance of knowing the kinetics can be highlighted if it can directly predict how fast a certain immobilisation reaction will happen. It has been seen that the kinetics is the same regardless of the actual starting amount of protein as displayed in Fig. 7.

Figure 7 shows that regardless of the initial amount of protein, the initial kinetics of this reaction is the same. In the case of the reduced amount of protein, all protein was immobilised within the first half an hour while for the full amount of protein approximately the same amount was immobilised after half an hour but longer time was required for everything to get immobilised.

In order to estimate the increase in reaction a simple exponential decay model in the form of $y = y_{0} + A_{0} e^{ - \lambda t}$ has been applied, where $y_{0}$ and $A_{0}$ are constants, *t* is time in hours and λ is the decay constant.

In Table 3 the optimised reactions refer to the ones that were found to be the most efficient immobilisation reactions in this paper. In the case of IgG, the Formyl immobilisation has been referred to as the original while the Tresyl one has been referred to as the optimised reaction. The time savings accounted for here are for the optimised reactions to reach the same extent of immobilisation as the original reactions.

**Table 3:** The estimated decay constants, λ, for the various protein immobilisation reactions from Figs. 2, 3, 4, 5, 6 and 7

Protein	Decay constant, λ, (h^−1^)		Time to original immobilisation		Saving
	Original	Optimised	Original (h)	Optimised (min)	Percentage (%)
BSA	0.66	0.87	6	55	85
Catalase	0.57	2.28	6	24	94
Lysozyme	0.8	2.44	4	5	98
mAb	0.33	1.28	24	14	99
IgG	Linear (k): 0.86	Linear (k): 4.39	24	30	98

The original immobilisation time and savings has been calculated based on λ

It can be seen that all the immobilisation reactions could be optimised to save 85% of time. All of these reactions could take place within an hour and most of them within half an hour to save valuable time compared to original immobilisation protocols that indicate that the reaction needs to be left overnight.

### Immobilisation discussion

All the proteins used in this paper have different sizes from 14.3 kDa (lysozyme) to around 250 kDa (catalase), which means that for the same amount in mg immobilised more protein molecules of the smaller proteins are bound to the resin. Based on the cross-sectional surface area, more mass of the larger proteins is needed in order to achieve the same surface coverage as the smaller proteins (Hedberg et al. 2016). In addition the kinetics of the reactions are faster for the smaller proteins in terms of more protein molecules bound in a shorter time frame.

The protein immobilisation reactions investigated also seemed to be independent on the scale used, as long as the ratio of the amount of particles in relation to the volume of protein solution was the same and the protocol was followed. The immobilisation efficiency of all proteins have increased by at least 60% relative to the conditions used before by researchers in Table 1. These improvements were especially found to be the case when changing from the original resin to Tresyl. For all proteins except catalase, Tresyl was found to be the best resin. Tresyl is an activated resin that is highly reactive towards amine and thiol groups, which are often both available in proteins. The resin works best in the neutral and slightly alkaline environments around pH 7–9, which is suitable for most proteins too. The coupling leads to the formation of a highly stable secondary amine or thio-ether linkage.

The Chromatographic Media Catalogue provides information about the ligand density for the different resins. Toyopearl AF-Formyl has a ligand density of 60 µeq/ml, and for Toyopearl AF-Amino and Toyopearl AF-Tresyl it is 30 µmol/ml and 80 µmol/g respectively. Assuming that each ligand binds one protein for each resin and there would be no limiting factors such as space and accessibility of binding groups, all resins would be able to bind over 300 mg of lysozyme and approximately ten times that amount for mAbs. This analysis concludes that the ligand group surface concentrations are not the limiting factor in terms of levels of protein immobilised, which means that it is the immobilisation chemistry in terms of the active surface groups that have shown to be more favourable than the amino and formyl resins for most of the proteins investigated here.

### Tresyl immobilisation—potential reactions

The addition of NaBH_3_CN has shown to increase the immobilisation reaction rate for Tresyl and has enhanced the amount of lysozyme immobilised (Fig. 4a and b) during this time frame in spite of the manufacturer’s recommendations (Table 2) stating that there is no need for a coupling agent. In Formyl, NaBH_3_CN helps the immobilisation to occur by reductive amination as the reactive ligands on Formyl are aldehyde groups. However, this enhancement seems to happen for Tresyl even though Tresyl does not have aldehyde groups on the surface. Here, NaBH_3_CN is clearly seen to accelerate reaction rates for lysozyme as shown in Fig. 4b. Many of the immobilisation reactions here have been with the amino groups, which are often located on the surface of the proteins. Amine modification is also a very common method for coupling different molecules to proteins (Smith 2006). The reaction below shows the mechanism of the Tresyl surface group binding to amine groups.

**Fig. 8:**
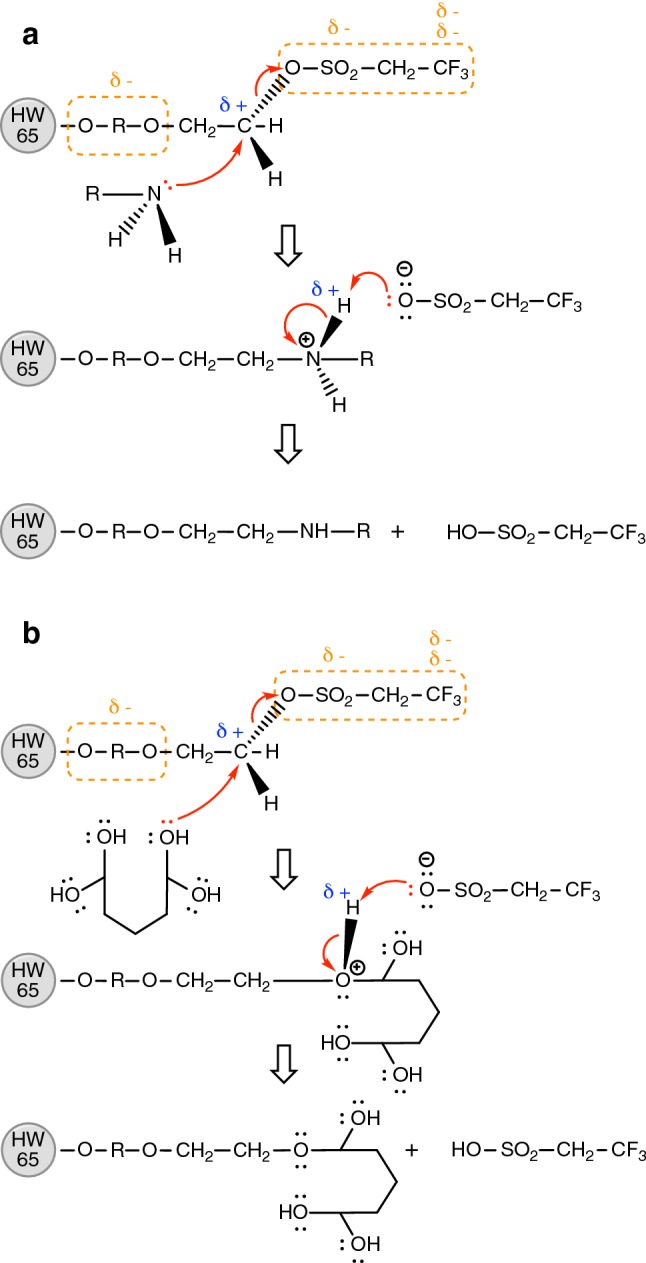
**a** The reaction mechanism of a spontaneous reaction between Tresyl particles and amine groups. HW 65 represents the Toyopearl AF-650M resin (without the surface group). **b** Proposed reaction mechanism between Tresyl particles and hydrated glutaraldehyde (cyclic hemiacetal). HW 65 represents the Toyopearl AF-650M resin (without the surface group)

During immobilisation, Toyopearl AF-Tresyl-650 M particles react spontaneously with amine and thiol groups present on the surface of the proteins. Thiol groups also possess a lone pair of electrons housed on an electronegative element (sulphur) that can undergo the same reaction. A nucleophilic substitution mechanism is proposed and illustrated in Fig. 8a. Amine groups contain an electronegative nitrogen atom capable of donating a lone pair of electrons to electrophilic centres. On the other hand, the Tresyl ligand contains several highly electronegative groups on either end which pull electron clouds away from the centre of the ligand. As a result, the central CH_2_ becomes an electrophile acquiring a strong partial positive charge. This allows the lone electron pair on the nucleophilic amine to attack the electrophilic carbon, resulting in the rest of the ligand (R-SO_3_^−^) to be eliminated. The R-SO_3_^−^ group can in turn abstract hydrogen from the positively charged nitrogen, resulting in the final immobilisation products.

Even though the addition of a coupling agent such as NaBH_3_CN or glutaraldehyde will both accelerate and enhance the reaction as can be seen in Figs. 4b and 5, glutaraldehyde exhibited superior success in immobilising BSA (Fig. 2), a mAb (Fig. 5) and a pIgG (Fig. 6). The exact structure and reaction mechanism of how glutaraldehyde binds to different molecules have been widely discussed, but it is still not completely understood. Nevertheless, it is well-known, widely used and one of the most effective cross-linker for proteins (Migneault et al. 2004). In terms of cross-linking protein this can take place either directly to a solid support (carrier) or between protein molecules (carrier-free). A common reaction is via the ε-amino group of lysyl residues (Weetall 1974). Lysyl ε-amino groups have a pKa of > 9.5, which means that there are some un-protonated forms of amine groups at lower pH’s sufficient to react with glutaraldehyde. It has been reported that at neutral or alkaline pH, glutaraldehyde may exist in a poly-glutaraldehyde forms that have different concentrations of aldehyde hydroxyl and carboxylic functional groups (Margel and Rembaum 1980). Glutaraldehyde can react with several functional groups of proteins such as amine, thiol, phenol, and imidazole (Habeeb and Hiramoto 1968). Therefore, there is an opportunity for it to react with many differing amino acid residues present in proteins.

Figure 8b displays a proposed reaction with the Tresyl resins to optimise the reactions towards a variety of different proteins. The above mechanism is commonly occurring among organic compounds that contain electronegative elements. Glutaraldehyde is another compound that contains electronegative components. According to Walt and Agayn (1994) glutaraldehyde forms various hydrated structures in aqueous form. One of these structures is a cyclic hemiacetal. The proposed hypothesis here is that this structure can potentially undergo the same nucleophilic substitution reaction as amine groups do as seen in Fig. 8a. However, due to its entropic structure and lower thermodynamic stability compared to the original R-SO_2_ chain, it is easier for the protein amine groups to be substituted (Fig. 8b). In other words, treatment of Tresyl particles with glutaraldehyde reduces the stability of the Tresyl ligand, hence increasing the immobilisation efficiency.

It has been experimentally verified that glutaraldehyde binds to the resin first by exposing a glutaraldehyde solution to resin and measuring the concentration after the exposure, which has then considerably decreased. This is also the mechanism for our immobilisation reactions, which means the resin is first activated with glutaraldehyde, then extensively washed so any residual glutaraldehyde not bound to the resin would not stay in the solution before any protein is introduced to the mixture. This eliminates any possibility that proteins would be cross-linked to other proteins with help of glutaraldehyde as would not be desirable for this purpose of adsorption. In this case we assume that all or most of the Tresyl surface groups have bound glutaraldehyde molecules and that the proteins exposed will react with the glutaraldehyde monolayer on the resin surface.

## Conclusion

In this paper the kinetics and extent of protein immobilised have been reported for a number of immobilisations reactions on commercial affinity chromatography resins. Optimisation of the solution reaction conditions and resins used for immobilising both model and therapeutic proteins are reported. In the new optimised conditions reported here using Tresyl resins with glutaraldehyde and NaBH_3_CN, the amount of protein immobilised was at least 60% higher than achieved with the immobilisation conditions most commonly reported in literature, and in the cases of lysozyme and a mAb up to 500% higher. It has also be seen that the optimised reactions have been able to save at least 85% of time from the originally recommended reaction times, and many of the immobilisations took place within half an hour. These time savings would be critical in an industrial setting where the output could be obtained much quicker, potentially incorporating future cost savings. The presence of NaBH_3_CN was found to significantly enhance reaction rates for some immobilisations reactions whilst glutaraldehyde allowed higher levels of protein immobilisation to be achieved. Finally, the proposed mechanisms for the optimised reactions have been illustrated to advance the understanding of the immobilisation reactions occurring.
